# Provision of a liquefied petroleum gas cookstove and fuel during pregnancy and infancy and linear growth trajectories between birth and 12 months: Evidence from the multi-center Household Air Pollution Intervention Network (HAPIN) trial

**DOI:** 10.1371/journal.pgph.0004831

**Published:** 2025-12-31

**Authors:** Kasthuri Sivalogan, Aryeh D. Stein, Lisa M. Thompson, Jiantong Wang, Anaite Diaz-Artiga, Vigneswari Aravindalochanan, Shirin Jabbarzadeh, Amy E. Lovvorn, Florien Ndagijimana, Laura Nicolaou, Kendra N. Williams, Kalpana Balakrishnan, Jennifer L. Peel, William Checkley, Thomas Clasen, Sheela S. Sinharoy, HAPIN investigators

**Affiliations:** 1 Nutrition and Health Sciences Doctoral Program, Emory University, Atlanta, Georgia, United States of America; 2 Hubert Department of Global Health, Emory University Rollins School of Public Health, Atlanta, Georgia, United States of America; 3 Family Health Care Nursing, University of California, San Francisco, United States of America; 4 Department of Biostatistics and Bioinformatics, Rollins School of Public Health, Emory University, Atlanta, Georgia, United States of America; 5 Center for Health Studies, Universidad del Valle de Guatemala, Guatemala CityGuatemala; 6 Department of Environmental Health Engineering, Indian Council of Medical Research Center for Advanced Research on Air Quality, Climate, and Health, Sri Ramachandra Institute of Higher Education and Research, Chennai, India; 7 Gangarosa Department of Environmental Health, Rollins School of Public Health, Emory University, Atlanta, Georgia, United States of America; 8 Eagle Research Center, Kigali, Rwanda; 9 Division of Pulmonary and Critical Care, School of Medicine, Johns Hopkins University, Baltimore, Maryland, United States of America; 10 Department of Environmental and Radiological Health Sciences, Colorado State University, Fort Collins, United States of America; 11 Center for Global Non-Communicable Disease Research and Training, School of Medicine, Johns Hopkins University, Baltimore, United States of America; Sustainable Futures Collaborative, INDIA

## Abstract

Exposure to particulate pollution from cooking with solid biomass fuels is associated with impaired child linear growth. We examined the effect of a liquefied petroleum gas (LPG) cookstove randomized controlled trial during pregnancy and infancy on linear growth trajectories among infants born to women enrolled during pregnancy. The Household Air Pollution Intervention Network (HAPIN) randomized controlled trial enrolled 3195 pregnant women (9 to <20 weeks’ gestation) from rural areas in Guatemala, Peru, India, and Rwanda who relied primarily on biomass fuels for cooking. Women in the intervention group received an LPG cookstove and fuel for approximately eighteen months, while those in the control group continued to use biomass for cooking. We measured the infants’ recumbent length at birth and 3, 6, 9 and 12 months of age and calculated length-for-age z-score (LAZ). We conducted a multiple group latent class growth analysis among the 2802 infants who finished the study and had ≥ 3 length measurements across the five timepoints to examine if latent classes differed by study arm. We identified three latent classes of linear growth, based on visual inspection of mean LAZ and model fit statistics, which represent higher, medium, and lower LAZ trajectories. Approximately 13.2% of infants belong to the high LAZ trajectory, 53.8% of infants belong to the medium LAZ trajectory and 33.0% belong to the low LAZ trajectory. The distribution of infants in each latent class did not differ by intervention assignment. Provision of an LPG cookstove and fuel during pregnancy and infancy did not alter linear growth trajectories among the offspring.

Clinical Trials Registration Number

ClinicalTrials.gov NCT02944682

## Introduction

Household air pollution, including from the incomplete combustion of solid fuels for cooking and heating using poorly ventilated combustion devices, continues to be a leading risk factor for global morbidity and mortality [1]. Women bear primary responsibility for cooking at the household level and therefore are at higher risk of cooking-related household air pollution exposure relative to other household members [2]. Women are also often primary caregivers for young children, and children’s physical proximity to their mothers during sensitive periods of growth and development between birth and two years of age may increase their own risk of household air pollution exposure [2,3]. Air pollution exposure is one of the leading threats to children’s health, accounting for almost 1 in 10 deaths in children under-five [4].

Prenatal household air pollution exposure can impair linear growth via oxidative stress and systemic inflammation, while postnatal exposure can result in impaired immune development and function, clinical and subclinical infection, dietary intake and metabolism and bone metabolism [3]. Risk of impaired linear growth is also associated with poor child development and increased risk for all-cause mortality, infectious disease mortality and chronic disease development in adulthood [3,5].

However, most of the evidence to date on household air pollution exposure and linear growth is derived from observational studies that report associations between estimated exposure at birth and growth outcomes [6]. A systematic review and meta-analysis of 11 studies with 168,298 children concluded that there was a 19% (95% CI: 1.10, 1.29) increased risk of stunting associated with exposure to household air pollution among children under five [7]. Most studies estimated household air pollution exposure based on self-reported fuel type and complementary determinants, including stove type, household ventilation and cooking habits (location, duration or frequency), without directly measuring exposure; furthermore, most studies were cross-sectional, with only one being a prospective cohort [7]. In addition, there is no multi-country evidence of household air pollution exposure on longitudinal growth patterns in the literature [8,9]. Additional evidence from longitudinal analyses is necessary to capture the rapid changes in growth that occur during early childhood.

The Household Air Pollution Intervention Network (HAPIN) trial was a randomized controlled trial (RCT) designed to assess the health effects of a liquefied petroleum gas (LPG) cookstove and fuel intervention compared to the continued use of biomass cookstoves. The trial had three primary outcomes focused on infants: low birth weight, severe pneumonia, and stunting at 12 months. As previously reported, birth weight of infants did not differ significantly between infants born to women in the intervention arm compared to those in the control arm [10]. Similarly, infants born to women in the intervention arm and infants born to women in the control arm had a similar mean length-for-age z-score (LAZ) at 12 months [8].

Here, we report the effects of the intervention on linear growth trajectories between birth and 12 months. The aim of this analysis is to describe longitudinal changes in LAZ among infants 0–12 months participating in the trial. Specifically, we examine patterns of growth by study arm, country, infant sex, and timing of intervention delivery.

## Methods

### Trial design and setting

HAPIN was a multi-center, parallel-group individual RCT to assess the effects of an LPG cookstove and fuel intervention on four primary outcomes: birth weight, stunting, and severe pneumonia in infants, and systolic blood pressure among older adult women (Clasen et al., EHP 2020). HAPIN investigators aimed to enroll 3200 pregnant women from four low- and middle-income countries (LMICs): Guatemala, India, Peru and Rwanda. Pregnant women were identified from clinical registries, prenatal clinics and/or referred by community health workers. Women were eligible if they were ages 18 to <35 years, cooked primarily with biomass stoves, lived in the trial area and were at 9 to less than 20 weeks’ gestation with a viable singleton pregnancy (confirmed by ultrasonography). Following written informed consent, and baseline surveys and assessments, pregnant women were randomized to the intervention or control group. Intervention households received a free LPG stove and fuel delivered to their household as needed for approximately 18 months (for the remainder of the pregnancy and through age 12 months of the infant), while control households continued to use their traditional biomass cooking fuel (principally wood, charcoal, and dung). Additional details of the intervention design have been described elsewhere [11].

### Ethics statement

The study protocol was reviewed and approved by institutional review boards or ethics committees at Emory University (00089799), Johns Hopkins University (00007403), Sri Ramachandra Institute of Higher Education and Research (IEC-N1/16/JUL/54/49), the Indian Council of Medical Research – Health Ministry Screening Committee (5/8/4–30/(Env)/Indo-US/2016-NCD-I), Universidad del Valle de Guatemala (146-08-2016/11–2016), Guatemalan Ministry of Health National Ethics Committee (11–2016), A.B. PRISMA (CE2981.17; CE2008.18; CE0028.20; CE0291.21), the London School of Hygiene and Tropical Medicine (11664), the Rwandan National Ethics Committee (No.357/RNEC/2018; 317/2017; 357/2018; 194/2019; 929/2020; 64/2021), and Washington University in St. Louis (201611159). Participants provided written informed consent. The trial is registered with ClinicalTrials.gov (Identifier NCT02944682; URL: https://clinicaltrials.gov/study/NCT02944682). The trial protocol (including planned primary and secondary outcomes), informed consent form, and primary outcome statistical analysis plans can be accessed at https://clinicaltrials.gov/study/NCT02944682. De-identified data associated with these analyses will be available through the Emory University Dataverse repository, linked to the DOI of this publication (https://doi.org/10.15139/S3/4WGVHC, Dataverse Link: https://dataverse.unc.edu/dataset.xhtml?persistentId=doi:10.15139/S3/4WGVHC. Other materials can be accessed upon request.

### Randomization and masking

Participants were randomly assigned in a 1:1 ratio stratified by setting (ten geographic strata from the four geographic regions of the trial: one district in Jalapa, Guatemala; two districts in Tamil Nadu, India; six provinces in Puno, Peru; and one district in Kayonza, Rwanda) in permuted blocks of two and four to either receive the intervention or continue their traditional cooking practices with biomass fuels. We sought to randomly assign 1600 participants each to both the intervention and control arms. Only one pregnant woman per household was allowed to participate. The HAPIN Data Management Core generated randomization lists based on block randomization using randomly selected block sizes, then prepared individual sealed envelopes containing trial group allocation, which were shipped to each study site and selected by participants. Due to the nature of the intervention, it was not possible to mask participants or data collection teams to the group assignment. However, the study investigators were blinded to the collected data, and primary analyses were conducted on blinded data. Participants in the control group received compensation, which varied by country, to offset the economic benefit of the intervention [12].

### Household air pollution exposure

Personal particulate matter less than 2.5 micrometers in diameter (PM_2.5_), black carbon (BC) and carbon monoxide (CO) exposure were measured three times during pregnancy – at baseline (9 to <20 weeks’ gestation, before randomization), between 24–28 weeks’ gestation, and between 32–36 weeks’ gestation [13]. Full details of the personal exposure assessment methods were previously described [13–15].

### Intervention fidelity and adherence; Exposure contrast between intervention and control groups

There was high fidelity and adherence to the LPG intervention overall [15,16]. In addition, there were large exposure contrasts in PM_2.5_, BC and CO between intervention and control arms at the first and second follow-up visits [14]. In the post-randomization follow-up period during pregnancy, 69% of intervention-group PM_2.5_ exposures were below the World Health Organization Annual Interim Target 1 compared to 23% of control-group PM_2.5_ exposures [14], indicating that the LPG intervention successfully reduced household air pollution exposures [14].

### Anthropometric outcomes

Following a standard protocol, recumbent length was measured twice to the nearest 1 mm with a Seca 417 measuring board (Seca, Hamburg, Germany) at five time points: birth (measured within 24 hours of delivery, typically at the health facility where they were born), and at home visits at 3, 6, 9 and 12 months of age (+/- 14 days). If the two length measurements differed by >0.7 cm, a third measurement was taken [17].

The COVID-19 pandemic resulted in some interruptions to in-home data collection, and specifically to anthropometric measurements. However, there were no differences in the proportion of infants with a missing length measurement between study arms before or after March 17, 2020 [8].

### Data management

Recumbent length was calculated as the average of the two closest measurements and converted to LAZ using the WHO Child Growth Standards SAS *igrowup* package [18]. Infants were included in the analysis if they had ≥ 3 length measurements across the five time points, to improve model stability [19]. Patterns of missing data by country, timepoint, study arm and infant sex are provided in the Supplemental Materials (S1 Table). Missing data are a minimal concern when missing at random in latent class models [20].

### Covariates

Maternal age, maternal education and maternal dietary diversity were assessed at baseline. Maternal education was categorized as: 1) no formal education or some primary school, 2) primary school or some secondary school, or 3) secondary, vocational or some university. Maternal dietary diversity was assessed using the tool adapted from the U.N. Food and Agriculture Organization to calculate Minimum Diet Diversity for Women [21]. Women were asked to recall the frequency of consumption of a prespecified list of food groups during the last 30 days at baseline and again at 6 months post-partum, which were then categorized into ten categories: grains, white roots, tubers, and plantains; pulses; nuts and seeds; dairy; meat, poultry, and fish; eggs; dark green leafy vegetables; other vitamin A-rich fruits and vegetables; other vegetables; and other fruits. Individual consumption was summed into a score ranging from 0-10 based on yes/no responses to the prespecified food groups and each woman was classified as achieving dietary diversity using a cut-off value of ≥5 [22]. Household food insecurity was assessed at baseline and classified according to the Food Insecurity Experience Scale [23]. Socioeconomic status was assessed at baseline and calculated as a derived index that incorporates water and sanitation quality, access to electricity, number of people in the household, food insecurity, participant’s education level, and type of floor, wall and roofing material.

### Statistical analysis

We used a multiple group latent class growth analysis to examine if patterns of linear growth from birth to 12 months of age across the 4 trial countries vary as a function of intervention arm (Model 1), controlling for the fixed effects of randomization strata [24]. Latent class growth modeling (LCGM) is a longitudinal analytical technique based on structural equational modeling that examines how individuals change by an observable outcome variable over time. In traditional growth modeling, it is assumed that all individuals in the study sample come from a single population and have one average trajectory to describe the underlying pattern of that single population. Individual differences are then captured by random slopes and random intercepts. In LCGM, individuals derive from multiple latent (or underlying) classes to identify distinct patterns within the study population. LCGM is a suitable approach for modeling repeated and highly correlated measures, such as early-life anthropometric measurements, and has a lower computational burden compared to growth mixture modeling [25,26]. In contrast to GEE or LMM, which assume a single underlying population trajectory and capture heterogeneity only through random effects, LCGA models unobserved heterogeneity by identifying latent subgroups. This is particularly relevant for repeated, highly correlated anthropometric measures in early life, where intervention effects may manifest differentially across subgroups of infants (e.g., those with persistently low LAZ vs. those showing catch-up growth). LCGA also provides a parsimonious, interpretable framework for describing these subgroups while retaining computational feasibility compared to growth mixture modeling [25,26].The multiple group analysis, using the “known class” function facilitates use of a grouping option to identity the variable in the dataset that contains information on group membership [27]. All models are adjusted for missing data under the assumption that data are missing at random using the full information maximum-likelihood (FIML) approach [28].

First, we plotted the trajectory of the mean LAZ score to verify longitudinal change and to identify the most appropriate pattern of change. Two measurements are needed to identify a linear model while three or more measurements are needed to identify a quadratic model. Then, we used individual LAZ scores at the five time points to identify the appropriate number of latent classes determined by model fit statistics, model parsimony and interpretability [26]. We used time scores (0–4) to reflect the five timepoints in our model because the timepoints were equidistant and to keep the quadratic time score smaller. We used Bayesian Information Criteria (BIC) to assess model fit and the Bootstrap Likelihood Ratio (BLRT) and Lo, Mendell, and Rubin Likelihood Ratio tests (LMR-LRT) to determine the number of latent classes [26]. We used entropy values to examine the separation of latent classes and the accuracy of classification into specific classes. Finally, we examined the posterior probabilities of an observation classified in each class to examine the percentage of the sample in each class [29]. We selected the final number of latent classes based on BIC, entropy, ability to make comparisons across different models, and not having small cell sizes in each class (<5%).

We also examined descriptive statistics by study arm and latent class trajectory group to assess if there were differences in sociodemographic characteristics by class membership.

All analyses were conducted in Mplus version 8 [30] and R version 4.2.2 [31]

### Subgroup analyses

We examined latent class growth trajectories separately by country (Model 2), infant sex (Model 3), and timing of the intervention delivery (Model 4) within the pooled sample. The multiple group analytical method allows class membership and model terms to vary by the variable of interest (e.g., by country) within each model. All subgroup analyses were prespecified. Due to the heterogeneous country settings, sub-analyses by country are standard for this trial. Additionally, there is evidence in the literature for heterogenous impact of household air pollution exposure on infant length by sex [9,32]. We also examined LAZ growth trajectories by sex separately for each country (Models 5–8). Prior analysis of the HAPIN intervention effect on birth weight suggested that infants of mothers who received the intervention prior to 18 weeks’ gestation had slightly higher birth weight than infants of mothers who received the intervention at 18 weeks or later [10]. Therefore, we examined whether LAZ growth trajectories differed by timing of the intervention (<18 weeks compared to ≥18 weeks) (Model 4).

We also controlled for the fixed effects of the randomization strata (trial sites within each country) in models examining latent class trajectories by infant sex and timing of intervention delivery in the pooled sample.

## Results

### Study population and participant characteristics

**Fig 1:**
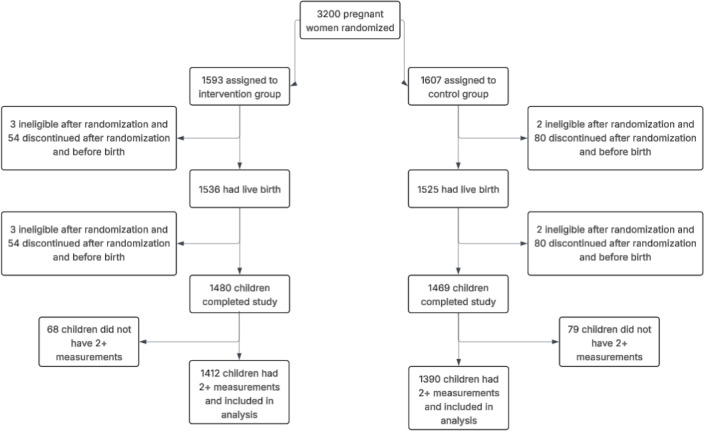
Final sample for analysis.

Between May 7, 2018, and February 29, 2020, 3200 pregnant women were randomized. Five participants were found to be ineligible after randomization, resulting in 3195 pregnant women participating in the trial. In addition, 4.2% (n = 134) exited the trial before giving birth and another 3.5% (n = 112) exited after the birth of the infant but before 12 months of age. Reasons for withdrawal or exiting the trial are provided in the Supplemental Materials (S2 Table). Harms and unintended events are described in Checkley et al., 2024 [8]. We excluded infants with two or fewer LAZ measurements across the five timepoints (n = 147), as indicated in S1 Fig. The final sample for this analysis included n = 2802 infants (Fig 1).

Descriptive characteristics for the analytical sample overall and by group are presented in Table 1. Descriptive characteristics by country are provided in the Supplemental Materials (S3 Table). Overall, pregnant women were enrolled at a mean gestational age of 15 weeks and reported consuming an average of three food groups on a daily basis. Women in both the intervention and control groups were of similar height and age. A higher percentage of women in the control group were nulliparous compared to women in the intervention group.

**Table 1:** Descriptive maternal statistics overall and by study group for analytic sample.

	Overall (n = 2802)	Intervention (n = 1412)	Control (n = 1390)
**Maternal height at baseline Mean (SD)**	152.19 (6.18)	152.24 (6.29)	152.13 (6.08)
**Maternal age at baseline (Mean, SD)**	25.41 (4.47)	25.34 (4.37)	25.48 (4.56)
**Gestational age at baseline (Mean, SD)**	15.39 (3.11)	15.52 (3.06)	15.26 (3.16)
**Nulliparous % (N)**	37.55 (1050)	35.52 (493)	39.56 (557)
**Socioeconomic index (Mean, SD)**	-0.01 (1.05)	0.05 (1.02)	-0.08 (1.07)
**Minimum dietary diversity (Mean, SD)**	3.41 (1.57)	3.46 (1.61)	3.37 (1.54)
**Household food insecurity (Mean, SD)**	1.38 (2.09)	1.28 (2.05)	1.48 (2.13)

### LAZ Latent Class Trajectories by ITT

**Fig 2:**
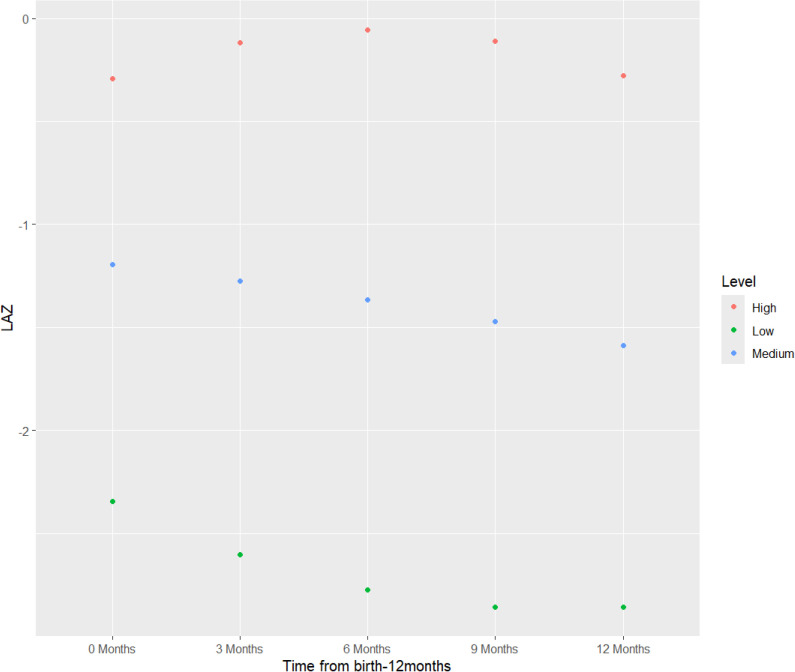
Latent classes for intention-to-treat analysis.

Mean LAZ at birth and at 3, 6, 9, and 12 months are presented in Table 2, and model fit statistics are presented in the Supplemental Materials (S4 Table). Log-likelihood and BIC decreased as the number of classes increased from a one to four class model (S4 Table). Entropy was > 0.80, indicating that latent classes are highly discriminating only for the three and four class models [33]. We determined that a three-class quadratic pattern of change best fit the LAZ data, based on visual inspection of mean LAZ and after evaluating the model fit statistics. We identified three LAZ latent class trajectories for both the intervention and control arms, as indicated in Fig 2.

**Table 2:** Observed mean LAZ of infants from birth to 12 months of age with at least three measurements.

	Overall (n = 2802)		Intervention (n = 1412)		Control (n = 1390)		Difference between Intervention and Control (95% CI)
	N	Mean (SD)	N	Mean (SD)	N	Mean (SD)	
**Birth**	2528	-1.01 (1.11)	1262	-0.98 (1.11)	1266	-1.05 (1.11)	-0.07 (-0.01, 0.15)
**3 Months**	2093	-1.16 (1.18)	1042	-1.13 (1.18)	1051	-1.18 (1.17)	-0.05 (-0.04, 0.14)
**6 Months**	2026	-1.10 (1.17)	1001	-1.11 (1.18)	1025	-1.08 (1.17)	0.03 (-0.12, 0.06)
**9 Months**	2036	-1.19 (1.15)	1014	-1.18 (1.15)	1022	-1.20 (1.14)	-0.02 (-0.07, 0.1)
**12 Months**	2267	-1.33 (1.14)	1138	-1.33 (1.16)	1129	-1.32 (1.12)	0.01 (-0.09, 0.07)

The three distinct latent class trajectory groups, with unique intercepts and slopes, are labelled as “high”, “medium” and “low”. The percentages of infants in each latent class were not significantly different between intervention (higher: 12.3% of infants, medium: 27.3%, lower: 10.8%) and control (higher: 10.6%, medium: 27.9%, lower: 12.1%) arms (p > 0.9). There was no overlap in intercepts or slopes between classes for either group. In addition, there were no significant differences in latent class LAZ growth trajectories between the intervention and control groups (p > 0.9) (Fig 2). For all three classes, the intervention group had a higher, but not significant, intercept and mean LAZ at all five timepoints as compared to the control group. Members of the high trajectory group in both intervention and control arms exhibited an increase in mean LAZ from birth to 6 months followed by a decrease from 6 to 12 months. Members of the medium and low trajectories in both intervention and control arms exhibited an overall decrease between birth and 12 months. Additionally, members of the low trajectory in both intervention and control arms had a mean LAZ less than 2 standard deviations (LAZ < -2 SD) from the mean from birth through 12 months of age.

We examined sociodemographic characteristics by study group assignment and latent class trajectory group (Table 3). In the intervention arm, there were differences in household socioeconomic index, maternal minimum dietary diversity score, maternal height, preterm births (birth that occurs before 37 weeks’ gestation), and the percentage of female infants by latent class trajectories. In the control arm, there were additional differences in household food insecurity score and the percentage of female infants.

**Table 3:** Descriptive statistics of study participants by LAZ latent class trajectories for 2802 infants with at least three measurements.

	Trajectory Group		
	High (N = 367)	Medium (N = 1507)	Low (N = 9256)
**Maternal height at baseline (Mean, SD)**	149.34 (6.11)	151.69 (5.93)	154.09 (6.07)
**Maternal age at baseline (Mean, SD)**	25.32 (4.61)	25.33 (4.50)	25.59 (4.35)
**Gestational age at baseline (Mean, SD)**	15.51 (3.13)	15.29 (3.08)	15.51 (3.15)
**Nulliparity (Yes) (N, %)**	117 (34.11)	580 (37.76)	353 (38.50)
**Infant sex (Female) (N, %)**	116 (33.72)	746 (48.50)	492 (53.48)
**Preterm birth (Yes) (N, %,)**	56 (16.28)	59 (3.84)	21 (2.28)
**Socioeconomic index (Mean, SD)**	-0.37 (1.03)	-0.04 (1.04)	0.17 (1.02)
**Minimum dietary diversity (Mean, SD)**	3.03 (1.42)	3.35 (1.56)	3.66 (1.62)
**Household food insecurity (Mean, SD)**	1.67 (2.31)	1.35 (2.07)	1.32 (2.03)

### LAZ Latent class trajectories by country and sex

**Fig 3:**
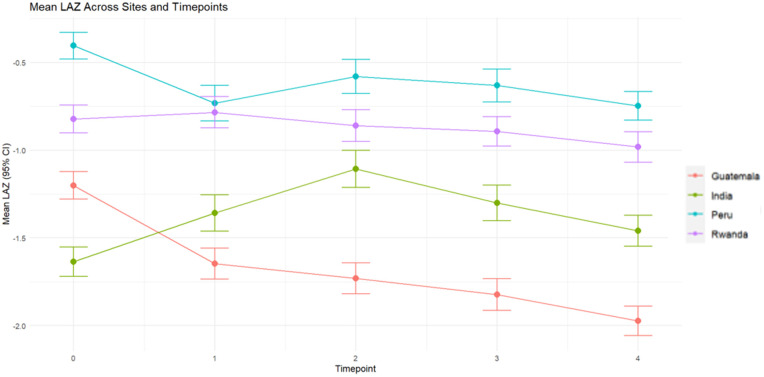
Mean length-for-age z-score (LAZ) by country and across all timepoints.

Individual latent class trajectories varied across countries between birth and 6 months of age, whereas trajectories were more consistent between 6 and 12 months of age. Infants, in both study arms, from all four countries had a mean LAZ below 0. Infants in Peru had the highest mean LAZ at all time points followed by those in Rwanda. Infants in India had the lowest mean LAZ at birth across all four countries, but infants in Guatemala had the lowest mean LAZ at all remaining timepoints. Notably, infants in all countries exhibited decreases in growth trajectories between 6 and 12 months, although rates of decrease varied by country site (Fig 3).

We used a multiple group latent class analysis to examine if patterns of linear growth vary as a function of country (Model 2) or infant sex (Model 3) within the pooled sample and by infant sex within each country site (Models 5–8). Model fit statistics for all subgroup analyses are presented in the Supplemental Materials (S6 Table). We did not identify any significant differences between latent class trajectories by country (p > 0.9) (Model 2) or by sex (p > 0.9) (Model 3). Additionally, there were no significant differences in latent growth trajectories by sex in three countries: India (p = 0.14), Peru (p = 0.12) or Rwanda (p > 0.9). However, we did identify significant differences in latent growth trajectories by sex specifically within the Guatemala sample (p < 0.0001) (Model 5). Guatemalan boys had lower mean LAZ, respective to Guatemalan girls, in all trajectory groups and at all timepoints.

### LAZ latent class trajectories by timing of intervention

Finally, we used a multiple group latent class analysis to examine if patterns of linear growth vary as a function of timing of intervention delivery during gestation. However, we identified no significant differences between those in the early and late gestation groups (p > 0.9) (Model 4).

## Discussion

In this study, we evaluated the impact of an LPG stove and free fuel intervention on linear growth trajectories during the first year of life in four LMICs. We identified three distinct latent class trajectories, consistent with the literature on linear growth trajectories [9,19,34]. In addition, the differing intercepts in the three-class model indicates that this heterogeneity is established prior to birth. A three-class model is both biologically and clinically significant, as it captures distinct growth patterns and highlights early-life variation that may have long-term implications. Despite high fidelity and adherence to the intervention and a large exposure contrast between study arms, we did not identify differences in latent class trajectories of LAZ growth between the intervention and control arms [14,16]. These results are consistent with previously published analyses of fetal growth, birth weight, and stunting at 12 months from this trial [8,10,35].

Our results on the differences in descriptive characteristics by class membership are consistent with the literature on known predictors of stunting. Maternal height and household wealth are known predictors of linear growth and are significantly associated with child stunting [36]. Preterm infants are underweight and stunted early in life, but they exhibit height and weight catch-up, primarily between 23–47 months of age [37,38]. These collective results do not support the use of environmental-specific interventions to improve child growth outcomes. Further analyses are necessary to examine linear growth patterns beyond 12 months of age, and to assess if patterns beyond 12 months change as a result of postnatal household air pollution exposure from birth to 12 months.

Our results differ from the prevailing observational literature that household air pollution exposure is associated with linear growth outcomes [7]. However, most of this literature relies on cross-sectional observational studies conducted in single countries, mostly in South Asia with some in sub-Saharan Africa. Most studies were primarily conducted in the postnatal period to examine the association of household air pollution exposure from solid fuel compared to cleaner fuel sources [7]. Observational household air pollution studies are also subject to uncontrolled confounding bias [39]. A few studies used longitudinal data and examined prenatal and postnatal exposure to household air pollution. The Ghana Randomized Air Pollution and Health Study (GRAPHS) examined the effect of both prenatal and postnatal household air pollution exposure on linear growth trajectories; similar to HAPIN, the cookstove intervention was not associated with linear growth trajectories [9]. However, GRAPHS reported that prenatal PM_2.5_ exposure was associated with impaired linear growth and increased stunting risk in the first year of life [9] based on a secondary exposure-response analysis with a limited number of participants in a single setting. And finally, the Randomized Exposure Study of Pollution Indoors and Respiratory Effect (RESPIRE) and follow-up Chronic Respiratory Effects of Early Childhood Exposure to Respirable PM (CRECER) cohort estimated that a 1ppm higher average carbon monoxide exposure was associated with a 0.21 lower height-for-age (HAZ), with the association being stronger among boys compared to girls [40]. Similarly to GRAPHS, the RESPIRE/CRECER intention-to-treat analysis indicated null results while the exposure-response indicated an effect on growth outcomes. Therefore, a next step will be to examine exposure-response relationships between PM_2.5_, BC and CO with patterns of LAZ growth in the HAPIN trial.

Interestingly, we only identified differences in latent growth trajectories by sex in Guatemala – for all latent class groups, the boys’ trajectory was consistently lower than the girls’ trajectory. These results are consistent with the literature on sex differences in linear growth – girls consistently have higher HAZ than boys [40–42]. However, it is unclear why these differences were only apparent in Guatemala. One explanation for the observed difference, specific to Guatemala, could be due to cultural perceptions of gender that result in different feeding behaviors. An ethnographic study conducted in an indigenous village in Guatemala highlighted that mothers reported introducing complementary foods early to infant boys because they perceived that breastmilk alone was not sufficient for infant boys [43]. Early introduction of complementary foods can lead to reduced consumption of breastmilk and its health-promoting factors which can increase exposure to illnesses that impair linear growth [43,44].

Finally, we did not identify differences in individual latent class trajectories by timing of the household LPG stove installation. Infants born to women who received the intervention at less than 18 weeks’ gestation (33.8g, 95% CI −2.6 to 70.2) had a slightly greater birth weight compared to infants born to women who received the intervention after 18 weeks’ gestations (5.3g, 95% CI -31.0 to 41.7g) [10]. However, results from both GRAPHS and HAPIN imply that the timing of the deployment of the intervention may not have been sufficient to address prenatal risk factors that can adversely affect birth and growth outcomes, including linear growth. GRAPHS identified sensitive windows in girls at 10 weeks’ gestation and in boys from 16-20 weeks’ gestation, where prenatal carbon monoxide exposure was inversely associated with birth weight [45]. The heterogeneity in the exposure reduction between the intervention and control arms in GRAPHS also suggest that reduction in household air pollution exposure must occur *prior* to the sensitive window periods [46]. While mechanisms underlying the timing of intervention delivery to linear growth or stunting outcomes are currently unclear, it has been proposed that first trimester interventions can have the largest effect on fetal growth ratio and birth length, and subsequently influence linear growth status and stunting [47,48]. Less than 2% (1.98%, n = 28) of pregnant women in HAPIN received the intervention in the first trimester (0–12 weeks) and thus, our results support earlier trial hypotheses that timing of intervention delivery beyond the first trimester may not affect linear growth trajectories.

Strengths of this study include the rigorous design, involving an RCT that was conducted in four geographically diverse regions with nearly exclusive adherence to the intervention and low attrition (10.8-13.5%) [8,14,16]. In addition, this analysis used high-quality repeated anthropometric measurements collected at ≥3 timepoints from over 2,800 infants between birth and 12 months of age. And finally, latent class methods facilitated the identification of latent subgroups of growth by country and timing of intervention, as well as to account for missing data using maximum likelihood under the missing at random assumption.

However, we acknowledge some limitations of this work. Firstly, there were interruptions to anthropometry data collection due to COVID-19 restrictions. As previously described, there were notable differences between intervention and control infants with missing measurements during the period of COVID-19 restrictions [8]. For example, intervention infants who were missing the length measurement at 12 months were born to mothers who were younger and taller and lived in households with less food insecurity than control infants who were also missing the length measurement at 12 months [8]. Secondly, some participants have missing data; we did not use multiple imputation, similar to the primary HAPIN outcome papers [10]. However, we did use the maximum likelihood estimator in Mplus with the assumption that missing data were missing at random. As noted above, fewer than 2% of pregnant women were enrolled in the first trimester and this constrains our ability to assess the impact of early prenatal exposure reductions on growth. And finally, although we were able to identify distinct classes of heterogenous latent growth in our data, latent class analysis assumes fixed variances within class and does not allow for within-class variation. There is also a risk that LCGM can introduce selection bias or reduce statistical power due to uneven group sizes and classification error.

Our study offers a comprehensive examination of child linear growth trajectories between birth and 12 months of age, a critical period that is sensitive to prenatal exposures and associated with growth and development beyond the first year of life. This analysis uses the most recent longitudinal data, from multiple time points that were collected in a standardized manner and from multiple global regions, to allow examination of child linear growth using both pooled and country-specific data. Our results corroborate the current school of thought from gold standard household air pollution - that an LPG cookstove intervention delivered after the first trimester of pregnancy is not independently sufficient to improve child linear growth outcomes between birth and 12 months of age.

## Supporting information

S1 Table Missing data for length-for-age z-score score measurements by timepoint, country and study arm assignment for 2802 children whose mothers completed participation in the HAPIN Trial. (TIFF)

S2 Table Reasons for withdrawal or exiting the HAPIN Trial. (TIFF)

S3 Table Descriptive characteristics by HAPIN country site for 2802 children whose mothers completed participation in the HAPIN Trial. (TIFF)

S4 Table Mean length-for-age z-score at each timepoint by intervention assignment and latent class trajectory for 2802 children whose mothers completed participation in the HAPIN Trial. (TIFF)

S5 Table Model fit statistics for latent class trajectories of length-for-age z-score by study arm for 2802 children whose mothers completed participation in the HAPIN Trial. (TIFF)

S6 Table Model fit statistics for latent class trajectories of length-for-age z-score by country, sex, timing of intervention and by sex within each country. (TIFF)

S1 Fig Path diagram of a longitudinal latent growth curve model. Observed variables are represented by squares and latent variables are represented by circles, with residuals represented by the small circles with an error term. The arrows between the latent variables, the intercept and slope, and the observed variables are fixed in advance. The factor loadings are fixed to 1 for the intercept latent variable and the factor loadings for the slope latent variable are fixed according to the change in time.
(TIFF)

S2 Fig Latent classes by study arm for intention-to-treat analysis. (TIFF)

S3 Fig Latent classes for timing of intervention delivery analysis. (TIFF)

S4 Fig Latent classes for sex analysis within Guatemala sample. (TIFF)
