# Supplementary figures and images for: Provision of a liquefied petroleum gas cookstove and fuel during pregnancy and infancy and linear growth trajectories between birth and 12 months: Evidence from the multi-center Household Air Pollution Intervention Network (HAPIN) trial
Source: PLOS Glob Public Health. 2025 Dec 31;5(12):e0004831. doi: 10.1371/journal.pgph.0004831 (PMC12755731; doi:10.1371/journal.pgph.0004831)

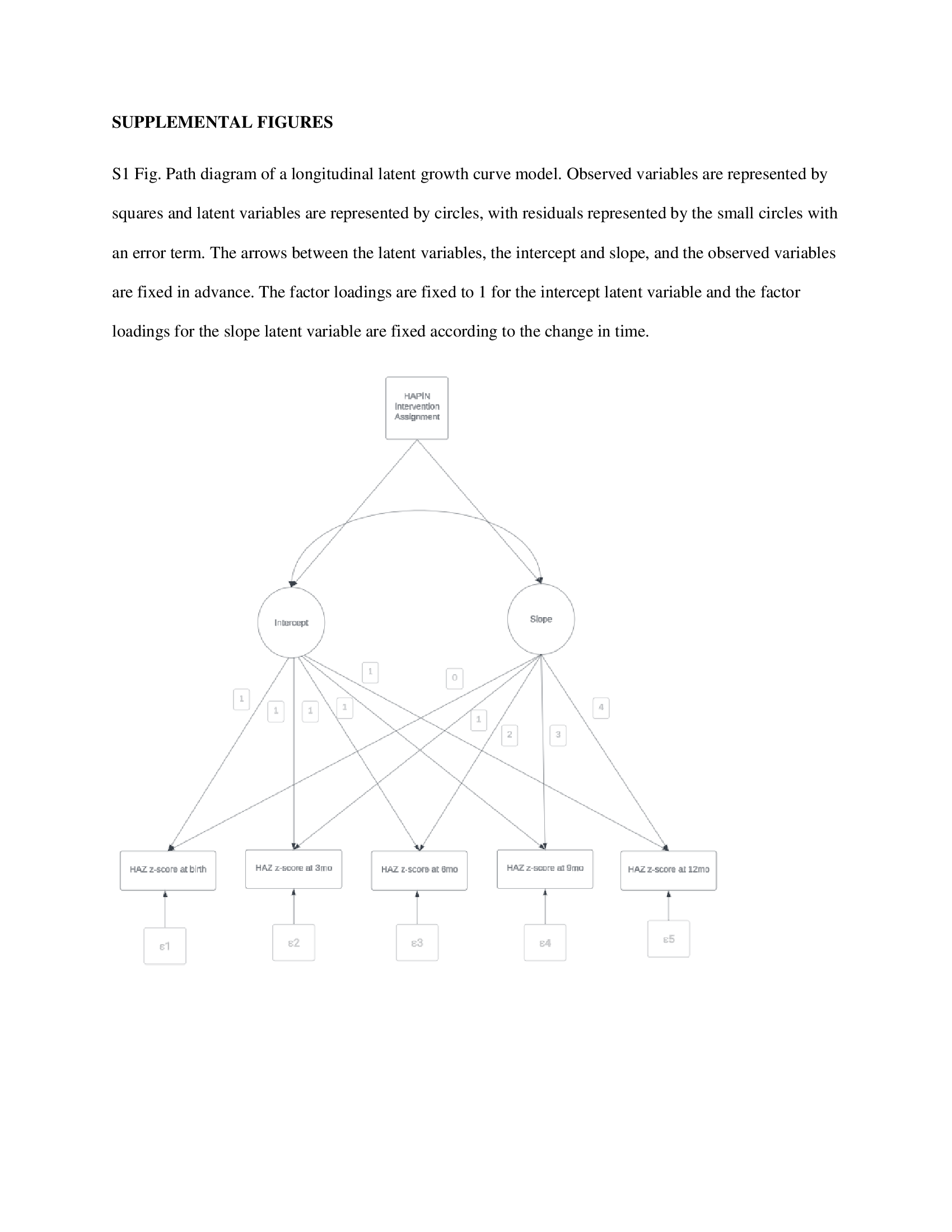

Supplement: S1 Fig — Observed variables are represented by squares and latent variables are represented by circles, with residuals represented by the small circles with an error term. The arrows between the latent variables, the intercept and slope, and the observed variables are fixed in advance. The factor loadings are fixed to 1 for the intercept latent variable and the factor loadings for the slope latent variable are fixed according to the change in time. (TIFF) [file pgph.0004831.s007.tiff]

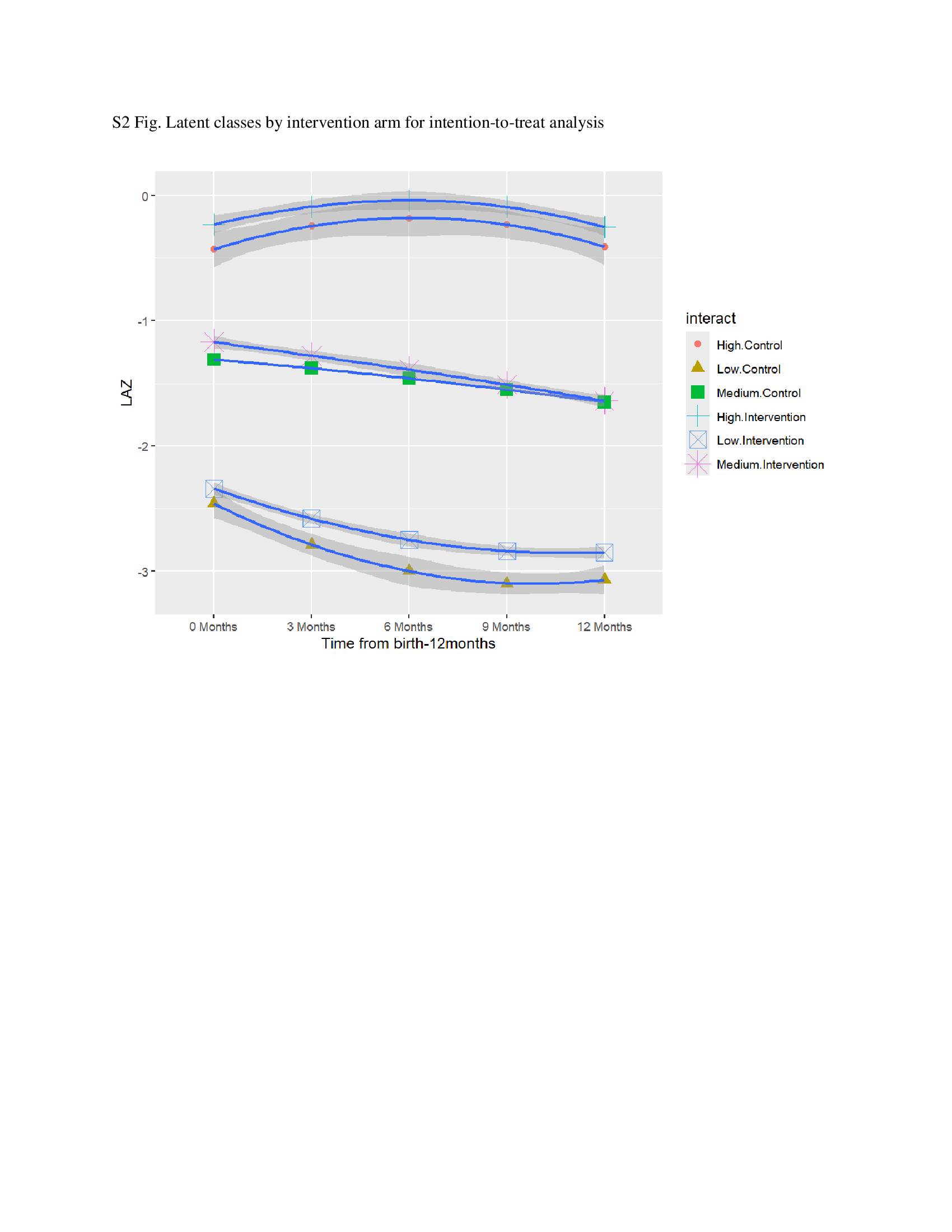

Supplement: S2 Fig — (TIFF) [file pgph.0004831.s008.tiff]

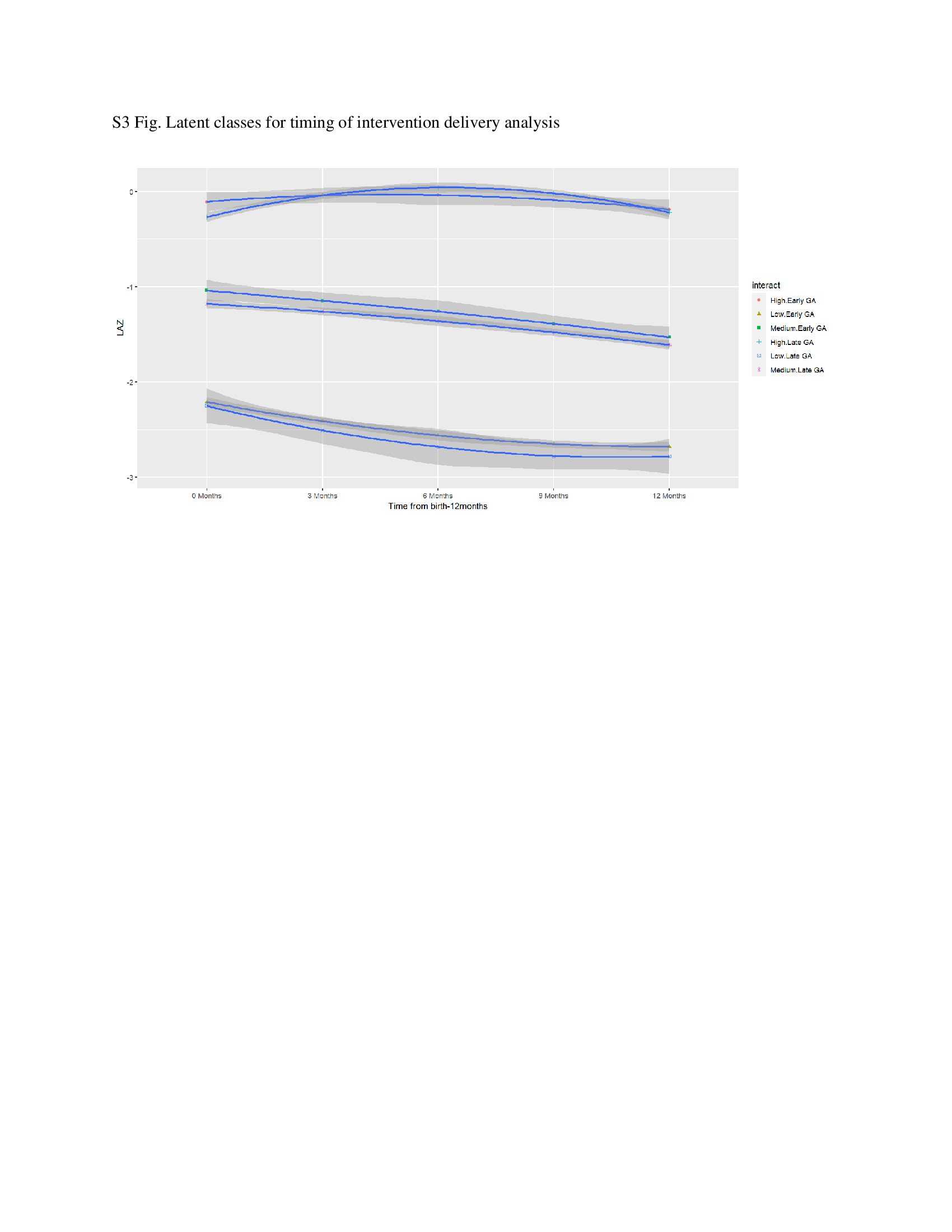

Supplement: S3 Fig — (TIFF) [file pgph.0004831.s009.tiff]

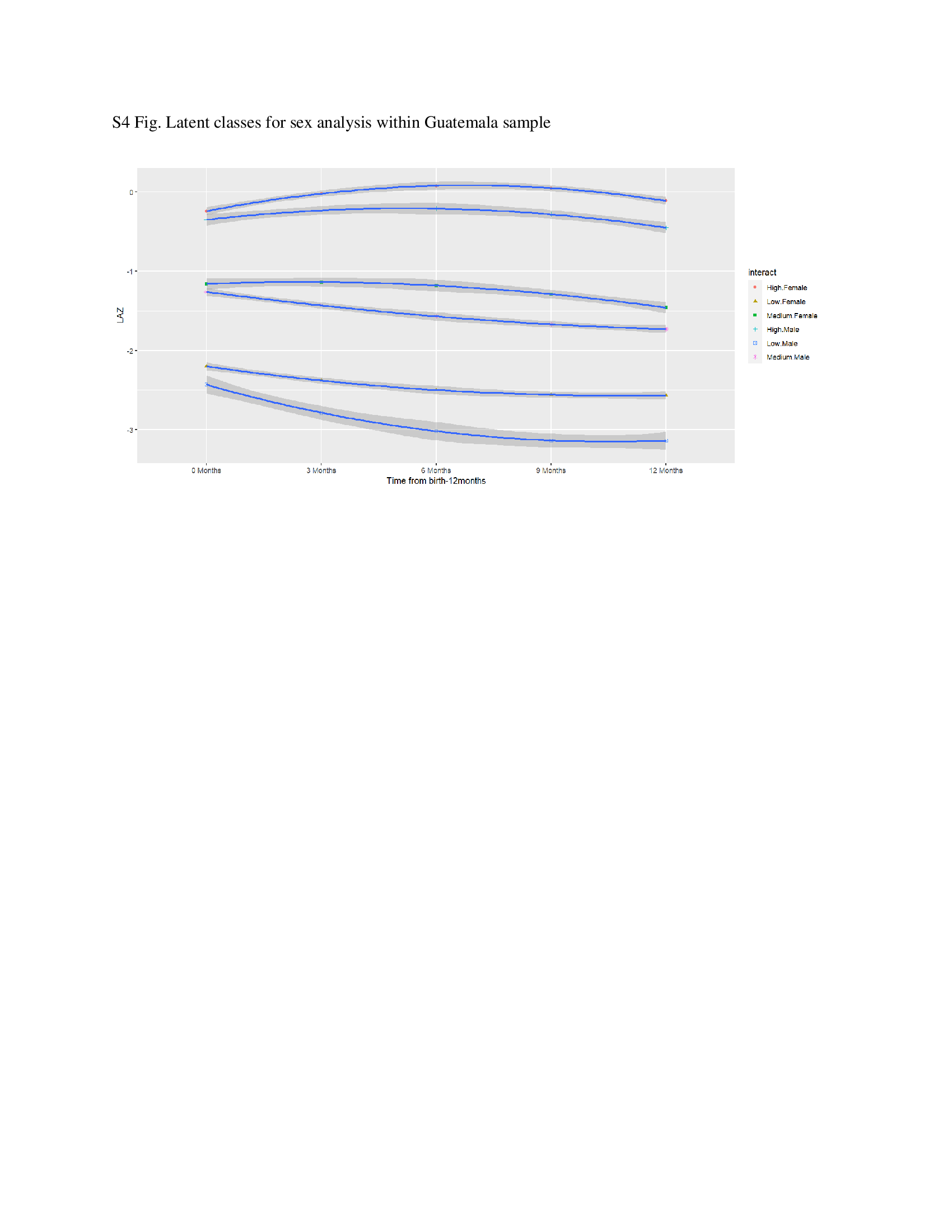

Supplement: S4 Fig — (TIFF) [file pgph.0004831.s010.tiff]
